# Sigmoid Colon Perforation, Pelvic Collection, and Hydronephrosis Caused by an Ingested Chicken Wishbone

**DOI:** 10.7759/cureus.52478

**Published:** 2024-01-18

**Authors:** Ali Yasen Y Mohamedahmed, Hassan Jouni, Jyotsna Kakarla, Mohamed Ebraheem, James Eccersley

**Affiliations:** 1 General Surgery, University Hospitals of Derby and Burton, Burton-on-Trent, GBR; 2 Colorectal Surgery and General Surgery, University Hospitals of Derby and Burton, Burton-on-Trent, GBR

**Keywords:** foreign body, chicken bone, sigmoid colon, hydronephrosis, large bowel perforation

## Abstract

This case report presents an unusual and challenging case of an 82-year-old female patient who presented with constipation and abdominal pain and was diagnosed with bowel perforation and hydronephrosis caused by an ingested chicken wishbone. This patient was treated with emergency laparotomy and bowel sigmoid resection. She made a good recovery and was discharged home. The patient's clinical presentation, diagnostic challenges, and successful management are discussed.

## Introduction

Bowel perforation is usually attributed to intrinsic or extrinsic obstruction due to tumours, adhesions, or ischaemia. Yet, it may be due to other factors such as foreign bodies. Most cases of ingested foreign bodies pass through the gastrointestinal tract without complications. However, the ingestion of bones, particularly fish or chicken bones, can lead to gastrointestinal perforation, with the sigmoid colon being one of the affected areas [1-6]. The diagnosis of bowel perforation caused by foreign bodies can be challenging due to its heterogeneous clinical symptoms (such as abdominal pain and constipation) and rarity. Fish and chicken bones are often difficult to visualise on X-ray, and computed tomography (CT) is essential for confirming the diagnosis and guiding surgical intervention [2,4]. Additionally, delayed bowel perforation can further complicate the diagnostic process, making it difficult to differentiate from other gastrointestinal pathologies [3].

We report a case of sigmoid perforation and hydronephrosis due to chicken bone ingestion. We aim to highlight the unusual presentation, diagnostic challenges, and management associated with this unusual cause of bowel perforation.

## Case presentation

**Figure 1:**
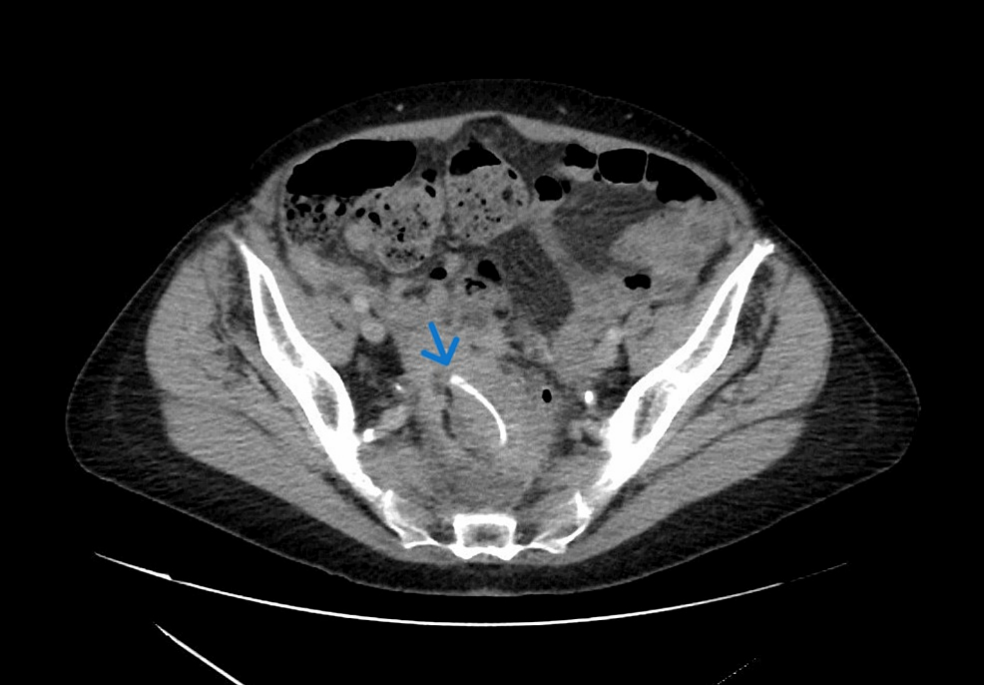
Abdomen and pelvis CT scan demonstrating the chicken bone (the blue arrow). CT: computed tomography.

**Figure 2:**
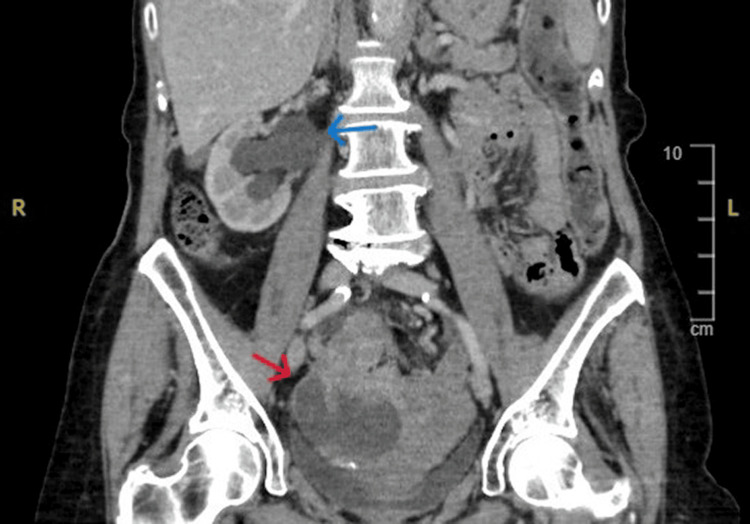
Abdomen and pelvis CT scan demonstrating right-side hydronephrosis (the blue arrow) and pelvic collection (the red arrow). CT: computed tomography.

An 82-year-old female presented to the emergency department at Queen’s Hospital Burton, United Kingdom, with recurrent episodes of constipation over four months. She was on different types of laxatives with minimal improvement in her symptoms. She denied rectal bleeding, weight loss, or relevant family history. The patient’s comorbidities included breast cancer, which was treated with surgery, radiotherapy, and hormonal therapy. Her abdomen was distended; however, there was no guarding or tenderness. Following admission, a CT scan with intravenous contrast of the abdomen and pelvis revealed a grossly thickened distal sigmoid colon with inflammatory changes, intraluminal hyperdensity, possibly representing an ingested animal bone, and local free fluid in the abdomen suggestive of a contained perforation (Figure 1). The differential diagnoses were malignancy, diverticulitis, infection, or traumatic bowel injury from the retained hyperdense object. The patient was treated conservatively with antibiotics and remained stable throughout the admission. Consequently, she was discharged with an outpatient flexible sigmoidoscopy. Three weeks later and before the sigmoidoscopy appointment, the patient presented to the emergency department with worsening symptoms, in particular abdominal pain and constipation. Abdominal examination showed a soft abdomen, and a blood test showed mild elevation of the inflammatory marker. The abdomen and pelvis CT scan showed similar findings to the previous scan, with pelvic collection causing right-side hydronephrosis (Figure 2).

**Figure 3:**
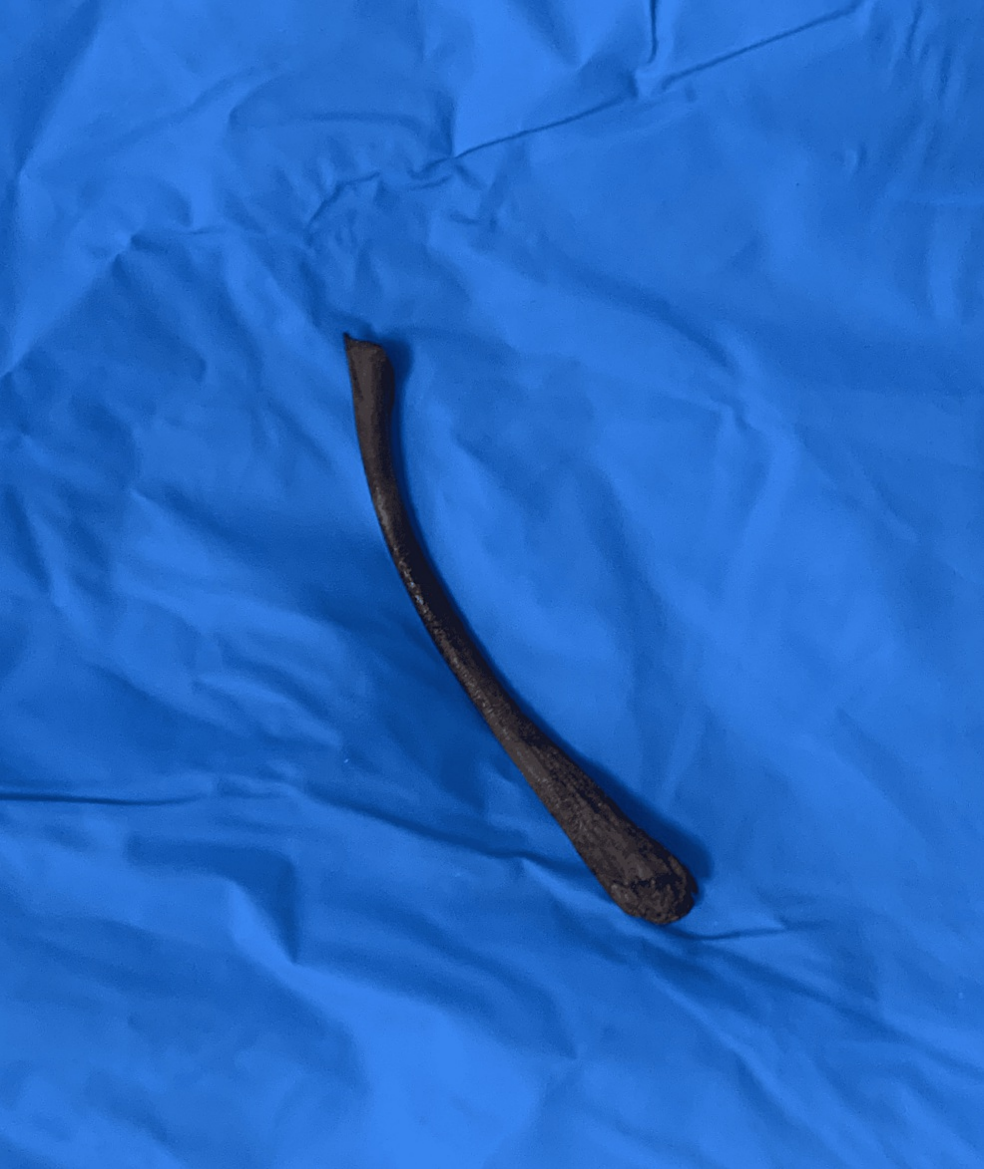
The retrieved chicken wishbone.

She was listed for emergency laparotomy, and the findings were dilated large bowels, obstructing fibrotic inflammatory mass in the sigmoid colon at the pelvic brim attached to the uterus. A chicken wishbone was found in the pouch of Douglas with a defect in the sigmoid colon wall (Figure 3). Sigmoid colectomy and primary stapler anastomosis were performed, and the patient made a good recovery and was discharged home.

## Discussion

Over 300 cases were reported in the literature of bowel perforation secondary to foreign bodies, with sharp bones such as fish bones being the commonest reason. The location of bowel perforation due to foreign body ingestion can vary, but there are specific sites that are more commonly affected. According to several studies, the most common sites of perforation due to ingested foreign bodies are the ileum, the rectosigmoid colon, and the narrowest parts of the bowel, such as the ileocecal valve or the rectosigmoid junction [2,7]. Diverticular disease predisposes the colon to be susceptible to perforation due to a foreign body, a chicken bone in our case. Patients may intentionally ingest the foreign bodies due to intellectual or mental problems, yet most patients who had unintentional foreign bodies ingested do not recall ingesting them, as was the case for the presented patient [8,9]. Moreover, it was described in the literature the accidental finding of colonic cancer in patients who had foreign body perforation, suggesting that the foreign body might get stuck due to the presence of the colonic mass [10].

During the first presentation of the above-reported case, the decision was to use conservative treatment and outpatient flexible sigmoidoscopy to visualise the bowel lumen, as the CT scan report raised the possibility of malignancy. In the second presentation, it was evident that the patient’s condition was not controlled with non-operative treatment. C-reactive protein and white blood cells were 81 mg/L and 11.8 10^*^9/L, respectively. Considering this and the mass effect of the collection, the decision was to perform an emergency laparotomy. The patient made a good clinical recovery as abdominal pain resolved, inflammatory markers improved significantly, and she passed bowel motion. No intervention was needed for the hydronephrosis as the cause was treated, and she was discharged on the eighth postoperative day. Moreover, the histopathology of the resected sigmoid colon showed multiple diverticulosis, inflammatory changes, and perforation in the bowel wall.

In cases of perforation caused by ingested foreign bodies, it is unusual to observe pneumoperitoneum on a simple abdominal film, as the object is often covered with fibrin and adjacent loops, preventing the passage of extensive quantities of fluid or gas into the peritoneal cavity [4]. CT plays a crucial role in diagnosing fish bone ingestion, as it is the most sensitive modality for detecting foreign bodies in the gastrointestinal tract [11].

Prompt and accurate diagnosis is crucial for initiating appropriate treatment and preventing further complications associated with foreign body ingestion [3,4]. Therefore, it is important to consider the possibility of foreign body ingestion when diagnosing patients with unexplained abdominal pain, especially in cases where there is a history of ingestion. Additionally, the type of foreign body, its shape, the location of impaction, the time elapsed since ingestion, and the presence of complications such as perforation, fistula, or bleeding are important factors in determining the management approach. Conservative management without intervention has been successful in 80% of ingested foreign body cases, especially when they are not associated with complications such as bleeding or perforation [12]. Endoscopic removal is recommended to prevent aspiration when the foreign body is lodged in the upper gastrointestinal tract, causing obstruction and the inability to manage secretions [13,14]. Moreover, colonoscopy has been utilized for visualising and extracting foreign bodies [14].

Surgical intervention is often necessary in cases of gastrointestinal perforation, with some cases requiring colectomy. Furthermore, the nature of the foreign body can also influence the management approach, as impacted foreign bodies may require careful inspection before intervention to exclude other causes of colonic obstructions [15]. The location of the foreign body within the colon can impact the decision-making process. For instance, the presence of a foreign body in the sigmoid colon with intestinal perforation has been documented, necessitating surgical management [15]. Furthermore, surgical treatment includes a wide range of modalities (bowel resection and anastomosis, Hartmann’s procedure, etc.) depending on the degree of abdominal contamination and involvement of other structures [2,15].

## Conclusions

Sigmoid colon perforation due to the ingestion of bone, particularly fish or chicken bones, is a rare but potentially serious complication. Moreover, it can be complicated with a collection that causes mass effect on other abdominal organs, such as the ureter in this case. Diagnosis can be challenging, and CT imaging is crucial for confirming the diagnosis and guiding surgical management. Given the potential for serious complications, prompt surgical intervention is often necessary in these cases.
